# Investigating the Antibacterial Ability of Sodium Hypochlorite Solution Activated with PUI and XPF File Against *Enterococcus faecalis* Using CFU, RT-PCR, and SEM

**DOI:** 10.3390/bioengineering11111086

**Published:** 2024-10-29

**Authors:** Radovan Jovanović, Jugoslav Ilić, Ljiljana Šubarić, Zoran Vlahović, Sanja Simić, Zoran Arsić, Milena Radunović, Branka Popović

**Affiliations:** 1 Dental Clinic, Faculty of Medicine, University of Priština in Kosovska Mitrovica, 38220 Kosovska Mitrovica, Serbia; ljiljana.subaric@med.pr.ac.rs (L.Š.); zoran.vlahovic@med.pr.ac.rs (Z.V.); zoran.arsic@med.pr.ac.rs (Z.A.); 2 Department of Restorative Odontology and Endodontics, School of Dental Medicine, University of Belgrade, 11000 Beograd, Serbia; jugoslav.ilic@stomf.bg.ac.rs; 3 Department of Microbiology and Immunology, School of Dental Medicine, University of Belgrade, 11000 Beograd, Serbia; milena.radunovic@stomf.bg.ac.rs; 4 Department of Human Genetics, School of Dental Medicine, University of Belgrade, 11000 Beograd, Serbia; branka.popovic@stomf.bg.ac.rs

**Keywords:** *E. faecalis*, endodontic, irrigation, infected canal, ultrasonic

## Abstract

Eradication of microorganisms present in the root canal system during endodontic therapy is one of the critical factors affecting the final outcome of endodontic treatment. However, even adequate technique of the root canal treatment and use of irrigants according to the established protocol does not lead to the complete elimination of microorganisms during endodontic treatment. The presence of *Enterococcus* (*E*.) *faecalis* inside the root canal system may be an important factor contributing to the failure of the treatment. Introducing agitation techniques in irrigation, such as passive ultrasonic irrigation (PUI) and the use of the activating instrument XP-endo Finisher (XPF), contribute to a better debridement and disinfection of the root canal. This study was conducted on 42 root canals experimentally inoculated with *E. faecalis* and exposed to three irrigation protocols. These have included a standard irrigation protocol using a syringe and needle, passive ultrasonic irrigation, and activation of the irrigant using an XPF instrument. The reduction in microorganisms was evaluated using the quantitative polymerase chain reaction in real-time (RT-PCR) as well as via the method of determining the number of colony forming units on nutrient medium (CFUs). The results of this study showed that the use of supplementary irrigation protocols, PUI, and XPF led to a significant decrease in the number of microorganisms inside the root canal of experimental teeth. These findings indicate a significant positive impact of these procedures on the disinfection of the root canal system.

## 1. Introduction

Eradication of microorganisms present in the root canal system during endodontic therapy is one of the critical factors affecting the final outcome [1,2,3]. The anatomical complexity of the root canal system as well as the resistance of bacterial biofilms result in the endodontic space only being adequately cleaned via mechanical activation of instruments. Removal of the pulpal tissue remains and the smear layer from the walls of the root canal can only be accomplished with the combination of irrigating solutions [4]. However, even the adequate technique of chemo-mechanical treatment of the tooth root canal according to the established protocol does not lead to the complete elimination of microorganisms from the root canal system [5,6] The standard irrigant solution used during endodontic therapy is sodium hypochlorite (NaOCl) [7]. Due to its antibacterial capabilities and tissue-dissolving abilities, it is considered the gold standard of irrigation in endodontics. The main means of NaOCl’s action is hydrolysis to form hypochlorous acid which further break down into new free oxygen that denatures bacterial proteins and has an antibacterial effect. The concentrations of NaOCl used in the clinic are 0.5%–8.25%. Over 2.5% NaOCl solution can dissolve organic tissues, and with the increase in concentration, temperature, volume, and time, the bactericidal effect and tissue dissolution ability are gradually boosted [8]. In addition to a large number of advantages that the use of NaOCl brings with it, there is a certain amount of disadvantage. The use of NaOCl in high concentrations yields a good antibacterial effect but also a high risk of complications such as extrusion beyond the apex [9]. With the use of a solution of NaOCl even in lower concentration with one of the agitation techniques, the antimicrobial effect may be increased and the risk of this complication may be significantly reduced [8,9]. Chlorhexidine (CHX) is used as an irrigant in endodontics due to its strong antibacterial effect against Gram-positive and Gram-negative bacteria and fungi. CHX in low concentrations increases permeability of the cell; the cell loses a large amount of potassium and phosphorus which causes bacterial death. In high concentration, it acts directly on the cytoplasmic part of the cell and causes death. The good side of CHX’s action is its prolonged bacteriostatic activity which can be retained in the root canal. However, CHX has no tissue-dissolving ability, so it cannot replace NaOCl in clinical practice.

In recent years, a vast number of additional antimicrobial procedures have been developed and introduced into everyday dental practice, which aim to eliminate residual microorganisms from the root canal system as efficiently as possible.

It is well known that one of the main reasons for endodontic failure is the presence of *Enterococcus* (*E*.) *faecalis* inside the root canal system. This finding can be explained by different factors, including its ability to form biofilms, invading dentinal tubules, and competition with other microorganisms leading to the resistance that this bacterium has demonstrated with the standard methods of removing bacteria from the tooth root canal [10,11,12].

Passive ultrasonic irrigation (PUI) is one of the most commonly used agitation techniques in irrigation [13]. The simplicity of the technique itself, as well as the availability of the equipment, has placed it at the very top of procedures in root canal irrigation. The ultrasound energy is transferred to the irrigant through an inactive instrument, which increases the depth of its penetration and its antimicrobial efficiency [14].

XP-endo Finisher (XPF-FKG Dentaire SA, La Chaux-de-Fonds, Switzerland) has been recently introduced as an instrument from a group of those that are aimed at increasing the efficiency of conventional irrigation. XPF is an ISO 25/00 instrument produced using a special type of alloy, the NiTi MaxWire (Martensite-Austenite Electropolish-FleX, FKG), and the file is straight in its M (martensite) phase when it is cooled, and it will change into the A (austenite) phase when it is exposed to body temperature where it will have its unique “spoon shape” with a length of 10 mm from the tip and a depth of 1.5 mm. Thanks to this construction, XP-endo Finisher effectively eliminates bacterial biofilm, primarily from the lumen of the main root canal [15].

## 2. Results

Results of our research show that there is a statistically significant decrease in the number of *E. faecalis* bacteria after the applied treatments in all experimental groups. The SEM analysis shows agreement with the findings of two previously applied methods for evaluating the effectiveness of antimicrobial procedures.

Statistical analysis of the obtained data on the number of *E. Faecalis* cells/RT-PCR depending on the applied therapeutic procedure yielded the following results (Table 1).

Statistical analysis of the obtained data on the number of colonies of bacteria *E. faecalis*/CFU, depending on the applied therapeutic procedure, yielded the following results (Table 2).

### SEM

**Figure 1:**
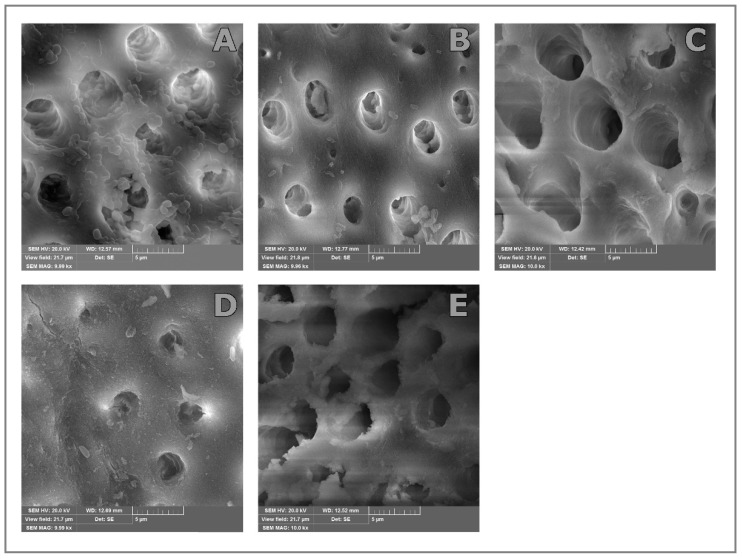
Scanning electron microscopy of dentin surface with *Enterococcus faecalis* (*E. faecalis*) biofilm under 10,000× magnification before irrigation protocol (**A**) and after the irrigation protocols: irrigation with saline (**B**); dentin surface after conventional needle irrigation (CNI) (**C**); passive ultrasound irrigation (PUI) (**D**); dentin surface after XP-endo Finisher (XPF) (**E**).

SEM images show a significant reduction in the number of microorganisms after the applied antimicrobial testing methods. In these images, the change in the structure of the dentine surface is also clearly visible (Figure 1).

A large number of bacterial cells can be seen on the SEM of the sample after induced infection with *E. faecalis* bacteria, at a magnification of 10,000 times. Bacterial cells are visible on the surface of the root canal, but at this magnification, penetration of bacteria is also visible in the dentin canals (Figure 1A).

SEM images of the tooth root canal surface after irrigation with saline (control group) shows a certain number of bacterial cells on the dentinal surface but also inside the dentinal canals (Figure 1B).

SEM images of the tooth root canal surface after CNI shows a significant reduction in bacterial cells on the dentinal surface. The noticeable phenomenon in this image is the presence of residual biofilm on the dentine surface (Figure 1C).

SEM images of the tooth root canal surface after PUI show a small number of bacterial cells. A partially eroded dentin surface with dentin fragments as well as crack formations in the peritubular dentin can be observed in Figure 1D.

The SEM image of the dentine surface after XPF treatment shows a small number of residual bacterial cells. Severe erosion of the dentine surface is observed, with partially or completely destroyed morphology of the initial part of the dentine tubules. A large amount of detritus is noticeable (Figure 1E).

## 3. Discussion

The success of endodontic treatment is conditioned by the effective eradication of microorganisms from the root canal space. This research was conducted in order to investigate the effectiveness of different antimicrobial procedures in eliminating *E. faecalis* as one of the most frequently isolated pathogens from the space of the tooth root canal. Due to its ability to penetrate the dentinal tubules and form a biofilm as well as its resistance to large ranges of pH values, this bacterium is known for its intense resistance to the effect of the common antimicrobial procedures in endodontic therapy [16,17]. Such findings can be explained by the fact that *E. faecalis* possesses the characteristic of coexistence with other bacteria as well as the ability to compete with them, penetrate deeply into the dentin canals, and survive in conditions of nutritional deprivation [18]. Irrigants used for root canal irrigation in endodontics have antibacterial properties and can decrease the number of microorganisms in the canal space. However, they have some disadvantages such as poor taste and smell, cytotoxicity, inability to remove the smear layer, as well as reduced antimicrobial efficiency in the presence of organic tissue, exudates, and bacterial plaques [19]. Therefore, in our study, we wanted to examine whether passive ultrasonic irrigation (PUI) and the use of XP-endo Finisher (XPF) increases the efficiency of the conventional irrigation procedure with NaOCl solution in eliminating *E. faecalis*.

The penetration depth of irrigants into the dentinal tubules is one of the dominant factors that determines the effectiveness of the elimination of microorganisms within the root canal space during endodontic therapy [20].

The PUI method aims to increase irrigant distribution by increasing the depth of its penetration within the root canals [21]. Gu et al. carried out a study to evaluate the penetration depth of irrigants into dentinal tubules by staining the NaOCl solution with fluorochromatic dyes, and by observing with a confocal laser microscope they concluded that PUI significantly increases the depth of irrigant penetration after its agitation with ultrasound [22].

Ultrasonic waves carry energy which can cause irrigant cavitation leading to effective biofilm removal from the root canal and its transfer to the lumen of main canal from where it is easier to eliminate [23].

In our research, PUI was applied for 60 s continuously, in contrast to the study carried out by Jussaro Alves Duque et al. who in their study applied PUI in three intervals of 20 s each with the addition of fresh NaOCl [24]. The addition of fresh NaOCl may affect its antimicrobial properties. In the study of Van der Sluis, when using PUI for 30 s, the use of ultrasound for 30 s leads to a rise in temperature, due to the cavitation effect of ultrasound [25].

By determining the number of bacterial genomes, in our study, we determined the effectiveness of irrigation techniques with NaOCl alone as PUI and XPF. NaOCl is an organolytic agent that effectively dissolves hereditary material, and it is known that the total number of selected segments of DNA or RNA is determined using the RT-PCR technique. RT-PCR, therefore, provides information on the total number of residual elements of hereditary material, while the determination of CFU/mL evaluates the number of living bacteria that can form colonies. The discrepancy in the number of bacteria estimated using RT-PCR and CFU may be explained as a result of these differences in experimental methods.

In the available literature, is not much data on the antibacterial effectiveness of special instruments’ design, such as the XPF, with the aim of making irrigation more efficient.

XPF has an exceptional effect on the elimination of *E. faecalis* bacteria, which becomes especially important if we consider the accessibility of XPF and its simple usage. Analyzing the value of percentage changes in CFU/mL after treatment in our study after the application of XPF, we registered a decrease in the number of bacteria by 99.3%.

Our findings correlate with the results obtained by Sasanakul et al. who, based on their research, conclude that the use of XPF as a supplementary procedure leads to an increase in efficiency of conventional irrigation with NaOCl solution [26]. The aforementioned authors, however, mention that the use of XPF, although non-invasive, causes turbulent flow of the irrigant inside the canal, which can lead to the extrusion of irrigant into the periapex area.

XPF was inserted 1 mm shorter than full working length, for one minute. According to Leonardy et al., it should be used twice for thirty seconds so that the sodium hypochlorite can be replaced with fresh solution more often in order to more effectively eliminate microorganisms and obtain a lower malleability of the file as the consequence of a lower frictional heating [27].

Juan Pacheco-Yanes et al. in their research evaluated the distribution of NaOCl throughout the root canal system, including the difficult-to-reach parts, after using PUI or the XP-endo Finisher instrument [20]. In their study, the authors concluded that the use of XPF shows better results compared to PUI and the control group in promoting distribution of the irrigant.

This result may be explained by the present findings, with the XP-endo Finisher instrument increasing the filling and distribution of the irrigant throughout the canal system [28]. The clinical implication of erosion after the application of XPF is that it can lead to a clean canal surface, but extensive erosion can lead to a decrease in the mechanical properties of dentin [29]. XP-endo finisher was used for 1 min according to the manufacturer guidelines for the instrument. Furthermore, in the PUI protocol, activation of the irrigation solution was conducted for the same period that contributed to the standardization of the activation procedures used in this study. Having in mind the effectiveness of the procedure that we found using our protocol and the dentinal erosions caused by the instrument, it may be concluded that longer application would contribute mainly to propagation of scrubbing effect on dentin.

However, the SEM micrographs showed that the erosions caused by the activity of the XP-endo Finisher are located on the very surface of dentin, similar to those caused by final irrigation protocols that include use of EDTA subsequently to NaOCl solution. There is no evidence that these superficial changes increase the risk of fracture or that it is anything more than a morphological alteration [30]. It may be assumed that, at this level of evidence, they do not have clinical significance.

The obtained microscopic image of *E. faecalis* corresponds to the appearance of this cell described in the literature [31]. This indicates that an adequate methodology for preparing samples for SEM analysis has been selected. It is also important to point out the micrograph after using XPF, which indicates a significantly eroded dentine surface after the effect of this instrument. This shows that this technique, although very effective in increasing the antimicrobial efficiency of irrigation, has an aggressive mechanical effect on the dentin surface.

## 4. Materials and Methods

The sample size was predefined using data from previously published papers that were based on the investigation of the disinfection of root canals with similar methodology [24,32,33], and the sample size was subsequently confirmed using G power 3.1.9.2 software (Heinrich Heine Universität Düseldorf, Düseldorf, Germany). The defined set of input parameters for our survey was α = 0.05, power of the test = 0.8, effect size f = 0.25 (medium), number of groups was four, number of measurements was two, correlation among repeated measures = 0.3 (weak), and the non-sphericity correction ε = 1, in order to conduct ANOVA for repeated measures. The required total sample size was 48 (12 per group). Due to technical problems, six specimens allocated in the control group were excluded. Since the standard deviation of subsequent findings in the control group, both in the RT PCR and CFU analysis, were in the range of standard deviations of the experimental groups, we decided not to replace them and extend the size of control group, in order to preserve the effect of the simultaneous specimen preparation in the same conditions. Therefore, this study was conducted on forty-two freshly extracted single-rooted permanent intact teeth with closed apices, which were extracted for orthodontic or periodontal reasons. The teeth were then disinfected and stored in 0.9% normal saline solution at room temperature. Before being decoronized, the teeth were cleaned of periodontium remnants and deposits using an ultrasound machine. An NTI^®^ Superflex Diamond Disc (KERR GMB Biberach Deutschland, Biberach, Germany) is used for cutting the crown so the length of the teeth was 13 mm. The working lengths were determined by placing the endodontic instrument K-file #10 (Dentsply Maillefer, Tulsa, UK) into the root canal until it was just visible at the apical foramen and then subtracting 0.5 mm. Root canal preparation was conducted at or within 0.5 mm working length determined using the Race Sequence Kit (FKG Dentaire SA, La Chaux-de-Fonds, Switzerland) up to instrument size R3 (30/04) and endo motor X Smart (Dentsply Sirona, Charlotte, NC 28277, USA) under NaOCl 2.5% irrigation. Elimination of the smear layer, compromising the subsequent contamination of dentine tubules with *E. faecalis* bacteria, was performed by immersing the specimens in a 17% concentration of EDTA solution (D line OU Kauplus Tallinn, Siauliai, Estonia) in a vortex apparatus for 4 min. Then, they were rinsed with 0.9% NaCl and treated in a vortex in a solution of 2.5% NaOCl for another 4 min and finally rinsed with 0.9% NaCl. In this way, the smear layer was completely removed. The teeth were individually wrapped for autoclaving and placed in an autoclave (121 °C; 15 min) for sterilization.

The strain of *E. faecalis* (ATCC 29212) (ATCC LGC Standards GmbH Mercator str. 51 46485 Wesel Germany) in a frozen state was grown and cultured on Broth Hewitt Todd agar plates for a period of 24 h at 37 °C. The single colonies were then transferred into dextrose broth and incubated at 37 °C for 24 h. Subsequently, the microbial suspension was prepared in dextrose broth with a concentration of microorganisms of 1.5 × 108 CFU/mL, with a density of 0.5 McFarland. Then, 20 µL of this suspension was injected into each canal individually with a micropipette. Specimens with inoculated microorganisms were placed into sterile Eppendorf tubes and incubated at 37 °C in order to ensure the attachment of bacteria to the dentin surface. Every 48 h, 20 µL of dextrose broth was added to ensure the exponential bacterial growth, after which the specimens were returned to the thermostat for incubation. After 14 days the incubation was stopped, and the 42 teeth were divided into three experimental groups and one control group.

In group I (12 roots), the standard tooth irrigation protocol was conducted using a syringe and needle/CNI (30 g; side perforated) with 2.5% NaOCl solution (pH 12), at room temperature (21 °C), in a volume of 5 mL. After the final irrigation, the residual NaOCl was inactivated with 1 mL of 5% sodium thiosulfate; then, the canals were irrigated with 1 mL of saline solution.

In group II (12 roots), passive ultrasonic irrigation/PUI was conducted with a Tigon W&H device (W&H Dentalwerk Bürmoos GmbH, Postfach 1, 5111 Bürmoos, Austria). After inserting 2.5% NaOCl (pH 12) into the canal, its activation was performed with an endodontic tip TYPE 1E for 60 s. After the final irrigation, the residual NaOCl was inactivated with 1 mL of 5% sodium thiosulfate; then, the canals were irrigated with 1 mL of saline solution.

In group III (12 roots), the canals were filled with 2.5% NaOCl solution and then treated with the XPF instrument (FKG Dentaire SA, La Chaux-de-Fonds, Switzerland) at a speed of 1000 revolutions per minute (rpm) and with 1 Ncm torque for 1 min. The residual NaOCl was inactivated with 1 mL of 5% sodium thiosulfate, and the canals were irrigated with 1 mL of saline solution.

In the control group (six roots), the teeth were rinsed with 5 mL of saline solution. 0.9% NaCl solution/Hemofarm/Serbia).

Before each antimicrobial protocol, the canals were irrigated with 2.5 mL of 0.9% saline solution which was additionally activated in the canal by inserting a #25 H-file instrument.

Two paper points were inserted serially into the canal up to the working length for 10 s. The paper points were then transferred to sterile Eppendorf tubes containing 1 mL RTF (Reduced Transport Fluid) solution and were sent for microbiological evaluation. The teeth were subjected to appropriate treatment after which the samples were taken again in the same way. The quantification of microorganisms was performed using the quantitative polymerase chain reaction in real-time (RT-PCR) as well as with the method of determining the number of the colony forming units on nutrient medium (CFUs).

Isolation of bacterial DNA was performed with a bacterial DNA isolation kit (PureLink Genomic DNA Mini Kit, Life Technologies, (Invitrogen, Carlsbad, CA, USA)) according to the manufacturer’s instructions. The assessment of bacterial DNA is expressed in the number of gene sequences for 16S rRNA per ml of sample (gc/mL) (gene copies per ml of sample). Amplification of the universal 16S rDNA sequence was performed using the SensiFASTtm SYBR^®^ Hi-ROX kit (Bioline, London, UK). The total volume of the reaction mixture for a single qPCR reaction was 15 μL and contained the following components: 7.5 μL SYBR Green qPCR Master Mix, 0.3 μM primers-EuF, EuR (1 μL each), 3.5 μL water, and 2 μL bacterial DNA. The temperature profile of the qPCR reaction was as follows: 95 °C for 10 min; 40 cycles, 95 °C for 1 min, 60 °C for 1 min, and 72 °C for 90 s; 72 °C for 5 min. The qPCR reaction was performed in a Line Gene Quantitative Fluorescent PCR Detection System (BIOER Technology Co., Hangzhou, China). After the qPCR reaction was completed, melting curve analysis was performed to confirm the specificity of each reaction. Also, each sample was analyzed twice. To assess the representativeness of the total number of rRNA genes in the analyzed samples, a reference bacterial strain, *Prevotela melaninogenica* (ATCC 25845), was used to obtain a standard curve. Based on the database http://www.ncbi.nlm.nih.gov/genome (accessed on 9 April 2018), the 3.17 Mb genome of *P. melaninogenica* was converted to a weight of 3.47 fg (the weight of one copy of the genome). Assuming that one genomic copy represents one bacterial cell and that *Prevotela spp.* have two rDNA operons for 16S rRNA, a standard dilution series was made from 32.4 fg DNA (20 16S rDNA genes) to 32.4 ng DNA (2 × 107 16S rDNA genes). To estimate the number of copies of the 16S rRNA gene, in the study samples, standards of known concentrations were included in each qPCR reaction, with a dilution factor of 10X. The analysis of the obtained values was conducted in Line Gene K software FQD-48A (Hangzhou BioerTechnology Co., Shanghai, China) (https://www.bio-equip.cn/enshow1equip.asp?equipid=1664, assessed on 25 September 2024). The number of gene copies of each sample was determined by comparison with the number of gene copies of the standard (16S rDNA genes), that is, based on the correlation between Ct values and known gene copies of the standard. Primer sequences were Fwd 5′GTTTATGCCGCATGGCATAAGAG 3′ and Rv 5′CCGTCAGGGGACGTTCAG 3′.

Two tenfold dilutions were made from the sampled liquid, and 20 µL of the bacterial solution of 1:100 dilution was seeded on blood agar (Himedia, India). The seeded nutrient media were incubated for 24 h at 37 °C. After a period of time, the number of colonies that have formed is counted for each sample. The total number of colonies is multiplied by the dilution factor (5000) in order to calculate the number of bacteria (CFU) per sample.

A single tooth from the experimental group was randomly selected, before and after treatment, for SEM analysis. The teeth were sectioned longitudinally with a high-speed fissure bur and then split in half with a dental chisel. The sample was fixed in 8% formaldehyde at 4 degrees C and stored for up to seven days. Dehydration was carried out via serial immersion in ethyl alcohol solutions of increasing concentrations (25%, 50%, 75%, 95%, and twice in absolute alcohol) for 1 h. The samples were prepared by steaming in an Au-Pd steamer and exposed to scanning electron microscopy at a magnification of 10,000 and 20,000 times. The analysis was conducted with a scanning electron microscope TESCAN FE-SEM Mira #XMU, Brno Czech Republic. Before treatment, SEM analysis of the samples confirmed biofilm formation and the presence of residual bacteria on the dentine surface and tubules after treatment.

### Statistical Data Processing

Descriptive statistical methods, methods for testing statistical hypotheses, and methods for evaluating agreement between methods of measurement were used to analyze the obtained data. Descriptive statistical methods were arithmetic mean (As), median (Med), measures of variability (standard deviation and interval). The method for testing statistical hypotheses was ANOVA with Tukey post hoc test. Statistical hypotheses were tested at a statistical significance level (alpha level) of 0.05. All data were processed in the IBM SPSS Statistics 22 (IBM Corporation, Armonk, NY, USA) software package.

## 5. Conclusions

The results of this study indicate that the use of supplementary irrigation protocols, i.e., agitation of irrigants, leads to a significant decrease in the number of microorganisms inside the tooth root canal during endodontic therapy of an infected canal. This result of the use of ultrasound and specially designed instruments affects the final outcome of endodontic therapy, making it more effective. The application of these supplementary root canal irrigation protocols is especially important if you consider the fact that these procedures are non-invasive, simple to implement, do not require special training or expensive equipment, and have a significant positive impact on the final outcome of endodontic therapy.

## Figures and Tables

**Table 1:** The number of bacterial cells obtained via the RT-PCR technique before and after the application of tested antimicrobial methods. CNI (conventional needle irrigation), PUI (passive ultrasonic irrigation), XPF (XP-endo Finisher), and Control (control group). *p* (statistical significance), max (sample maximum), min (sample minimum), med (median), s (sample standard deviation), and m (mean). The method for statistical testing: ANOVA with Tukey post hoc test.

Treatment		m	s	Med	Min	Max	*p*
CNI	Before	3.75 × 10^7^	4.72 × 10^7^	2.30 × 10^7^	1.13 × 10^6^	1.32 × 10^8^	0.001
	After	9.82 × 10^4^	9.80 × 10^4^	8.38 × 10^4^	6.70 × 10^3^	3.45 × 10^5^	
PUI	Before	1.35 × 10^7^	1.25 × 10^7^	8.94 × 10^6^	4.50 × 10^5^	3.48 × 10^7^	0.001
	After	5.86 × 10^4^	4.39 × 10^4^	4.15 × 10^4^	1.11 × 10^4^	1.46 × 10^5^	
XPF	Before	3.07 × 10^5^	7.09 × 10^5^	1.03 × 10^5^	1.82 × 10^4^	2.55 × 10^6^	0.001
	After	7.82 × 10^3^	9.81 × 10^3^	3.93 × 10^3^	7.90 × 10^2^	3.58 × 10^4^	
Control	Before	1.74 × 10^7^	3.50 × 10^7^	1.72 × 10^6^	6.30 × 10^5^	8.00 × 10^7^	0.001
	After	4.10 × 10^6^	7.78 × 10^6^	2.26 × 10^5^	1.40 × 10^5^	1.80 × 10^7^	

**Table 2:** The value of CFU/mL before and after the application of the investigated antimicrobial procedures. CNI (conventional needle irrigation), PUI (passive ultrasonic irrigation), XPF (XP-endo Finisher), and Control (control group). *p* (statistical significance), max (sample maximum), min (sample minimum), med (median), s (sample standard deviation), and m (mean). The method for statistical testing: ANOVA with Tukey post hoc test.

Treatment		m	s	Med	Min	Max	*p*
CNI	Before	1.50 × 10^6^	4.93 × 10^5^	1.80 × 10^6^	7.80 × 10^5^	1.98 × 10^6^	0.002
	After	1.70 × 10^5^	1.90 × 10^5^	1.05 × 10^5^	5.00 × 10^1^	6.80 × 10^5^	
PUI	Before	1.07 × 10^6^	2.76 × 10^5^	1.00 × 10^6^	7.00 × 10^5^	1.50 × 10^6^	0.002
	After	2.34 × 10^5^	2.40 × 10^5^	1.80 × 10^5^	8.00 × 10^1^	7.60 × 10^5^	
XPF	Before	1.96 × 10^5^	1.15 × 10^5^	1.85 × 10^5^	4.00 × 10^4^	4.00 × 10^5^	0.002
	After	2.33 × 10^3^	2.67 × 10^3^	2.00 × 10^3^	0.00. × 10^0^	9.00 × 10^3^	
Control	Before	1.66 × 10^6^	3.05 × 10^5^	1.55 × 10^6^	1.30 × 10^6^	2.00 × 10^6^	0.043
	After	9.60 × 10^5^	1.52 × 10^5^	8.70 × 10^5^	8.30 × 10^5^	1.15 × 10^6^	

## Data Availability

Dataset available on the request from the authors.

## References

[1] Plotino G., Colangeli M., Özyürek T., DeDeus G., Panzetta C., Castagnola R., Grande N.M., Marigo L. (2021). Evaluation of smear layer and debris removal by stepwise intraoperative activation (SIA) of sodium hypochlorite. Clin. Oral. Investig..

[2] Prada I., Micó-Muñoz P., Giner-Lluesma T., Micó-Martínez P., Collado-Castellano N., Manzano-Saiz A. (2019). Influence of microbiology on endodontic failure. Literature review. Med. Oral Patol. Oral Cir. Bucal..

[3] Siqueira J.F., Rôças I.N. (2022). Present status and future directions: Microbiology of endodontic infections. Int. Endod. J..

[4] Lane J., Bonsor S. (2019). Survival rates of teeth treated with bacterial photo-dynamic therapy during disinfection of the root canal system. Br. Dent. J..

[5] Siqueira Junior J.F., Rôças I.D.N., Marceliano-Alves M.F., Pérez A.R., Ricucci D. (2018). Unprepared root canal surface areas: Causes, clinical implications, and therapeutic strategies. Braz. Oral Res..

[6] Gomes B., Berber V.B., Kokaras A.S., Chen T., Paster B.J. (2015). Microbiomes of endodontic periodontal lesions before and after chemomechanical preparation. J. Endod..

[7] Haapasalo M., Shen Y., Wang Z., Gao Y. (2014). Irrigation in endodontics. Br. Dent. J..

[8] Zou X., Zheng X., Liang Y., Zhang C., Fan B., Liang J., Ling J., Bian Z., Yu Q., Hou B. (2024). Expert consensus on irrigation and intracanal medication in root canal therapy. Int. J. Oral. Sci..

[9] Guivarc’h M., Ordioni U., Ahmed H.M., Cohen S., Catherine J.H., Bukiet F. (2017). Sodium Hypochlorite Accident: A Systematic Review. J. Endod..

[10] Xu J., He J., Shen Y., Zhou X., Huang D., Gao Y., Haapasalo M. (2019). Influence of Endodontic Procedure on the Adherence of *Enterococcus faecalis*. J. Endod..

[11] Gaeta C., Marruganti C., Ali I.A.A., Fabbro A., Pinzauti D., Santoro F., Neelakantan P., Pozzi G., Grandini S. (2023). The presence of *Enterococcus faecalis* in saliva as a risk factor for endodontic infection. Front. Cell. Infect. Microbiol..

[12] Wang L., Dong M., Zheng J., Song Q., Yin W., Li J., Niu W. (2011). Relationship of biofilm formation and gelE gene expression in *Enterococcus faecalis* recovered from root canals in patients requiring endodontic retreatment. J. Endod..

[13] Nagendrababu V., Jayaraman J., Suresh A., Kalyanasundaram S., Neelakantan P. (2018). Effectiveness of ultrasonically activated irrigation on root canal disinfection: A systematic review of in vitro studies. Clin. Oral. Investig..

[14] Niavarzi S., Pourhajibagher M., Khedmat S., Ghabraei S., Chiniforush N., Bahador A. (2019). Effect of ultrasonic activation on the efficacy of antimicrobial photodynamic therapy: Evaluation of penetration depth of photosensitizer and elimination of *Enterococcus faecalis* biofilms. Photodiagn. Photodyn. Ther..

[15] Bao P., Shen Y., Lin J., Haapasalo M. (2017). In Vitro Efficacy of XP-endo Finisher with 2 Different Protocols on Biofilm Removal from Apical Root Canals. J. Endod..

[16] Yang S., Meng X., Zhen Y., Baima Q., Wang Y., Jiang X., Xu Z. (2024). Strategies and mechanisms targeting *Enterococcus faecalis* biofilms associated with endodontic infections: A comprehensive review. Front. Cell. Infect. Microbiol..

[17] Delboni M.G., Gomes B.P., Francisco P.A., Teixeira F.B., Drake D. (2017). Diversity of *Enterococcus faecalis* Genotypes from Multiple Oral Sites Associated with Endodontic Failure Using Repetitive Sequence-based Polymerase Chain Reaction and Arbitrarily Primed Polymerase Chain Reaction. J. Endod..

[18] Ramsey M., Hartke A., Huycke M., Gilmore M.S., Clewell D.B., Ike Y., Shankar N. (2014). The Physiology and Metabolism of Enterococci. Enterococci: From Commensals to Leading Causes of Drug Resistant Infection.

[19] Dioguardi M., Di Gioia G., Illuzzi G., Laneve E., Cocco A., Troiano G. (2018). Endodontic irrigants: Different methods to improve efficacy and related problems. Eur. J. Dent..

[20] Pacheco-Yanes J., Provenzano J.C., Marceliano-Alves M.F., Gazzaneo I., Pérez A.R., Gonçalves L.S., Siqueira J.F. (2020). Distribution of sodium hypochlorite throughout the mesial root canal system of mandibular molars after adjunctive irrigant activation procedures: A micro-computed tomographic study. Clin. Oral Investig..

[21] Ahangari Z., Asnaashari M., Akbarian Rad N., Shokri M., Azari-Marhabi S., Asnaashari N. (2021). Investigating the Antibacterial Effect of Passive Ultrasonic Irrigation, Photodynamic Therapy and Their Combination on Root Canal Disinfection. J. Lasers Med. Sci..

[22] Gu Y., Perinpanayagam H., Kum D.J., Yoo Y.J., Jeong J.S., Lim S.M., Chang S.W., Baek S.H., Zhu Q., Kum K.Y. (2017). Efect of diferent agitation techniques on the penetration of irrigant and sealer into dentinal tubules. Photomed. Laser Surg..

[23] Sheetal R., Barsha S., Sultan A., Omar K., Alfred T., Amr S.F. (2023). Evaluation of Antibacterial Efficacy of High-Intensity Focused Ultrasound Versus Photodynamic Therapy Against *Enterococcus faecalis*–Infected Root Canals. Ultrasound Med. Biol..

[24] Duque J.A., Duarte M.A.H., Canali L.C.F., Zancan R.F., Vivan R.R., Bernardes R.A., Bramante C. (2017). Comparative Effectiveness of New Mechanical Irrigant Agitating Devices for Debris Removal from the Canal and Isthmus of Mesial Roots of Mandibular Molars. J. Endod..

[25] Van Der Sluis L.W.M., Versluis M., Wu M.K., Wesselink P.R. (2007). Passive ultrasonic irrigation of the root canal: A review of the literature. Int. Endod. J..

[26] Sasanakul P., Ampornaramveth R.S., Chivatxaranukul P. (2019). Influence of Adjuncts to Irrigation in the Disinfection of Large Root Canals. J. Endod..

[27] Leonardi D.P., Dds N.M.G., Tomazinho F.S.F., Marques-Da-Silva B., Gonzaga C.C., Filho F.B., Plotino G. (2019). Influence of activation mode and preheating on intracanal irrigant temperature. Aust. Endod. J..

[28] Elnaghy A.M., Mandorah A., Elsaka S.E. (2017). Effectiveness of XP-endo Finisher, EndoActivator, and File agitation on debris and smear layer removal in curved root canals: A comparative study. Odontology.

[29] Mathew D.M., Durvasulu A., Shanmugam S., Pradeepkumar A.R. (2023). Evaluation of Different Agitation Techniques on Smear Layer Formation and Dentine Erosions—An In Vitro Study. Eur. Endod. J..

[30] Boutsioukis C., Arias-Moliz M.T. (2022). Present status and future directions—Irrigants and irrigation methods. Int. Endod. J..

[31] Bulacio M.D.L.Á., Galván L.R., Gaudioso C., Cangemi R., Erimbaue M.I. (2015). *Enterococcus faecalis* Biofilm. Formation and Development in Vitro Observed by Scanning Electron Microscopy. Acta Odontol. Latinoam..

[32] Leoni G.B., Versiani M.A., Silva-Sousa Y.T., Bruniera J.F., Pécora J.D., Sousa-Neto M.D. (2017). Ex vivo evaluation of four final irrigation protocols on the removal of hard-tissue debris from the mesial root canal system of mandibular first molars. Int. Endod. J..

[33] Căpută P.E., Retsas A., Kuijk L., Chávez de Paz L.E., Boutsioukis C. (2019). Ultrasonic Irrigant Activation during Root Canal Treatment: A Systematic Review. J. Endod..

